# Supplementary material for: Severe acute respiratory disease in American mink experimentally infected with SARS-CoV-2
Source: JCI Insight. 2022 Nov 22;7(22):e159573. doi: 10.1172/jci.insight.159573 (PMC9746805; doi:10.1172/jci.insight.159573)
Supplement: Supplemental data [file jciinsight-7-159573-s148.pdf]

Supplementary Materials

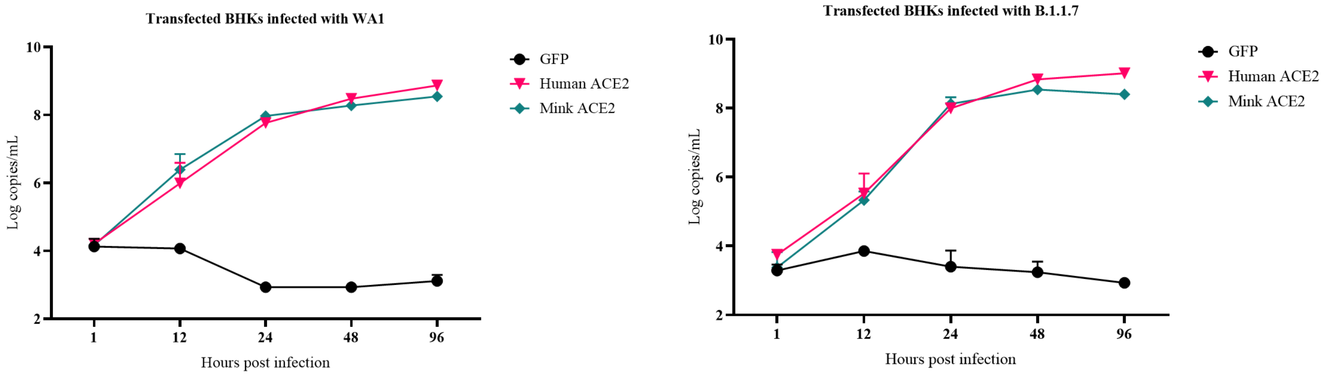

Supplemental Figure 1. Viral growth curves. Supernatant was collected from human and mink ACE2 and GFP transfected BHKs infected with either lineage A or B.1.1.7 SARS-CoV-2 variants at 1-, 12-, 24, 48-, and 96- hours post-infection in triplicate. Data are mean and standard error of the mean genomic RNA represented as log copies/mL

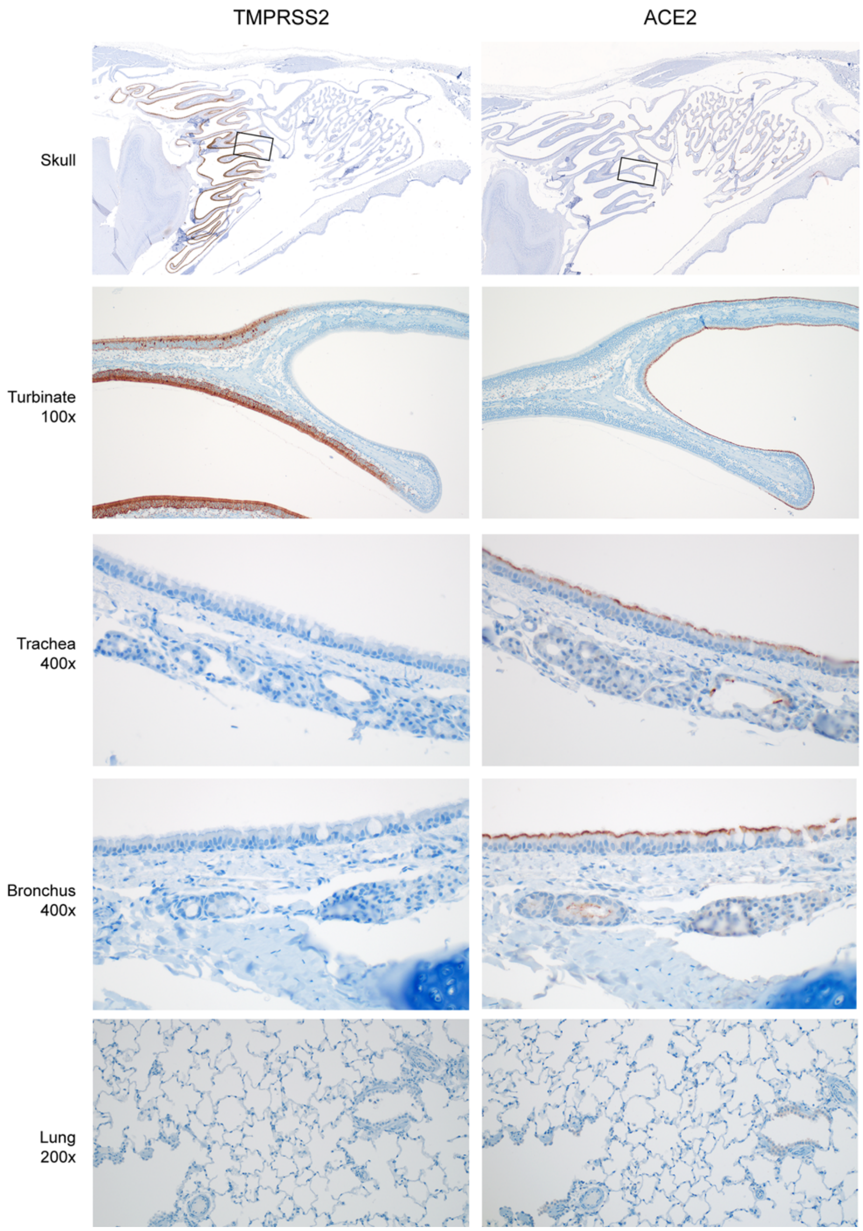

Supplemental Figure 2. Nasal turbinate olfactory and respiratory epithelial TMPRSS2 and ACE2 immunohistochemistry. Sagittal section of mink skull, TMPRSS2 (left column) and ACE2 (right column). Note the TMPRSS2 immunoreactivity of the caudal (olfactory epithelial) region of the turbinates. Higher magnification of the turbinate reveals TMPRSS2 immunoreactivity of the olfactory epithelium sharply demarcated from the ACE2 immunoreactivity of the respiratory epithelium lining the more rostral portion of the turbinate. The tracheal and bronchial epithelium are not immunoreactive for TMPRSS2 but are for ACE2; whereas the lung is immunoreactive for neither.

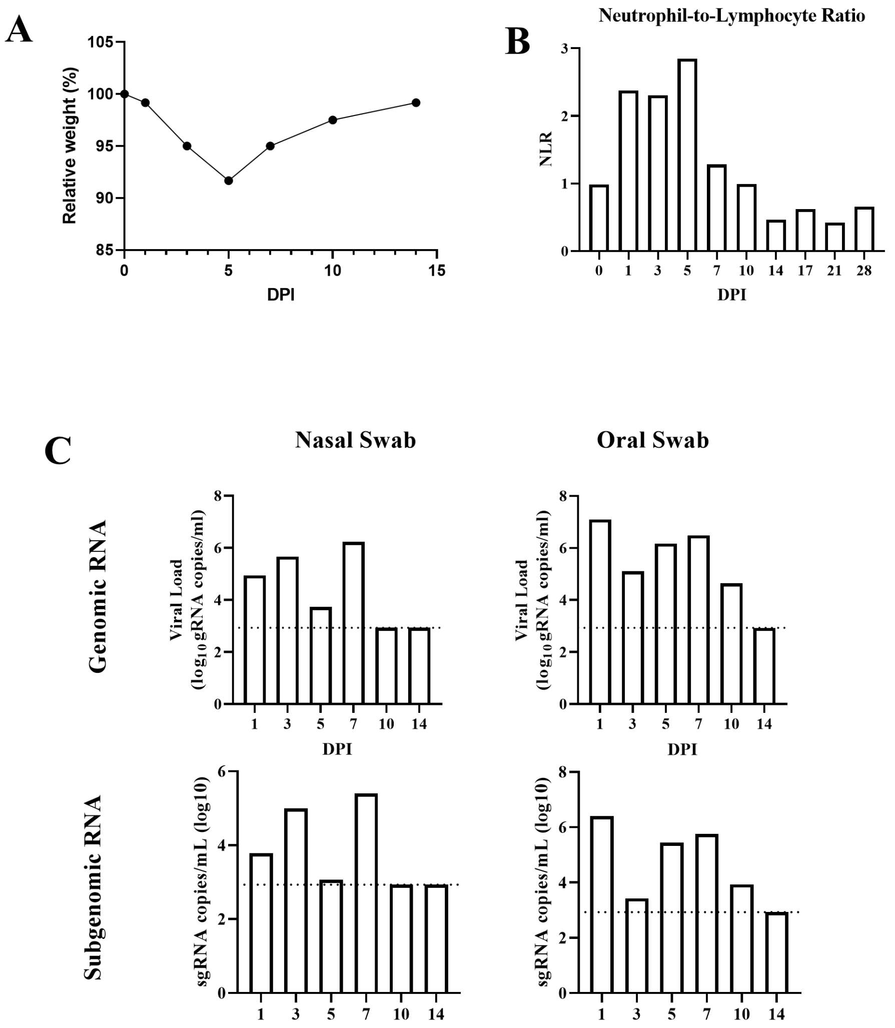

Supplemental Figure 3. Resolution of clinical disease in the surviving animal. The surviving animal was monitored for change in relative weight (A). Weight loss was most severe on 5 DPI, after which the animal began recovery. Neutrophil-to-lymphocyte ratio was monitored over time (B), with the most severe change appreciated on 5 DPI. Nasal and oral swabs were evaluated for resolution of viral shedding through genomic and subgenomic RT-PCR (C). All swabs on 14 DPI were below the limit of detectable virus.

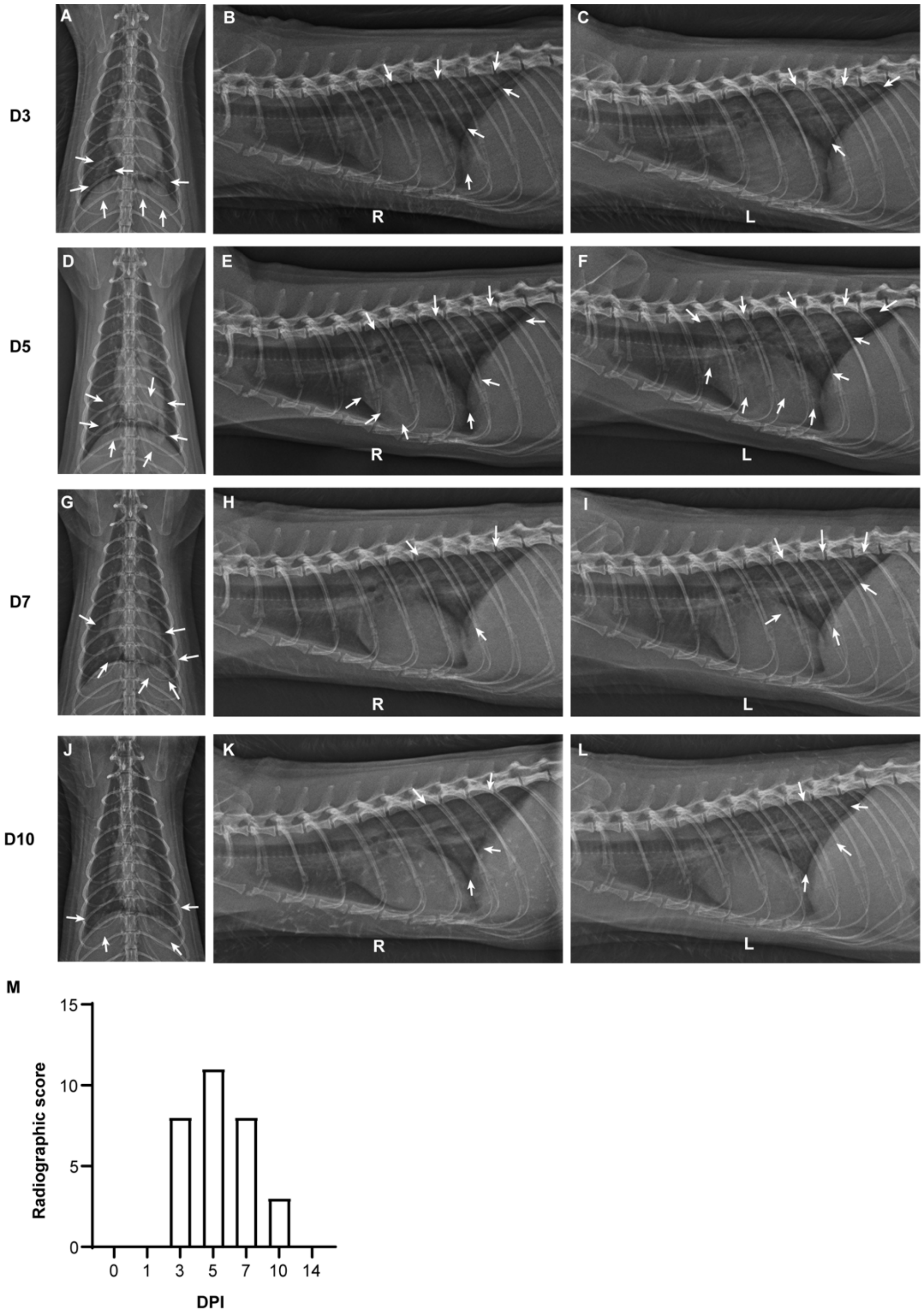

Supplemental Figure 4. Resolution of radiological disease in the surviving animal. (A-L) Dorsoventral, right lateral, and left lateral radiographs from surviving animal on 3, 5, 7, and 10 DPI. Arrows indicate pulmonary infiltrates, first visible in the left and right caudal lung lobes at 3 DPI (A-C) with additional involvement in the caudal subsegment of the left cranial lung lobe on 5 DPI (D-F). There is mild improvement in the alveolar pattern in the left and right caudal lung lobes on 7 DPI, with resolution in the caudal subsegment of the left cranial lung lobe (G-I). The pulmonary changes continued to improve by 10 DPI, with grade 2 pulmonary disease in the right caudal lung lobe with grade 1 pulmonary disease in the left caudal lung lobe consistent with improving viral pneumonia and pneumonitis (J-L).(M) Radiographic scores for surviving animal.

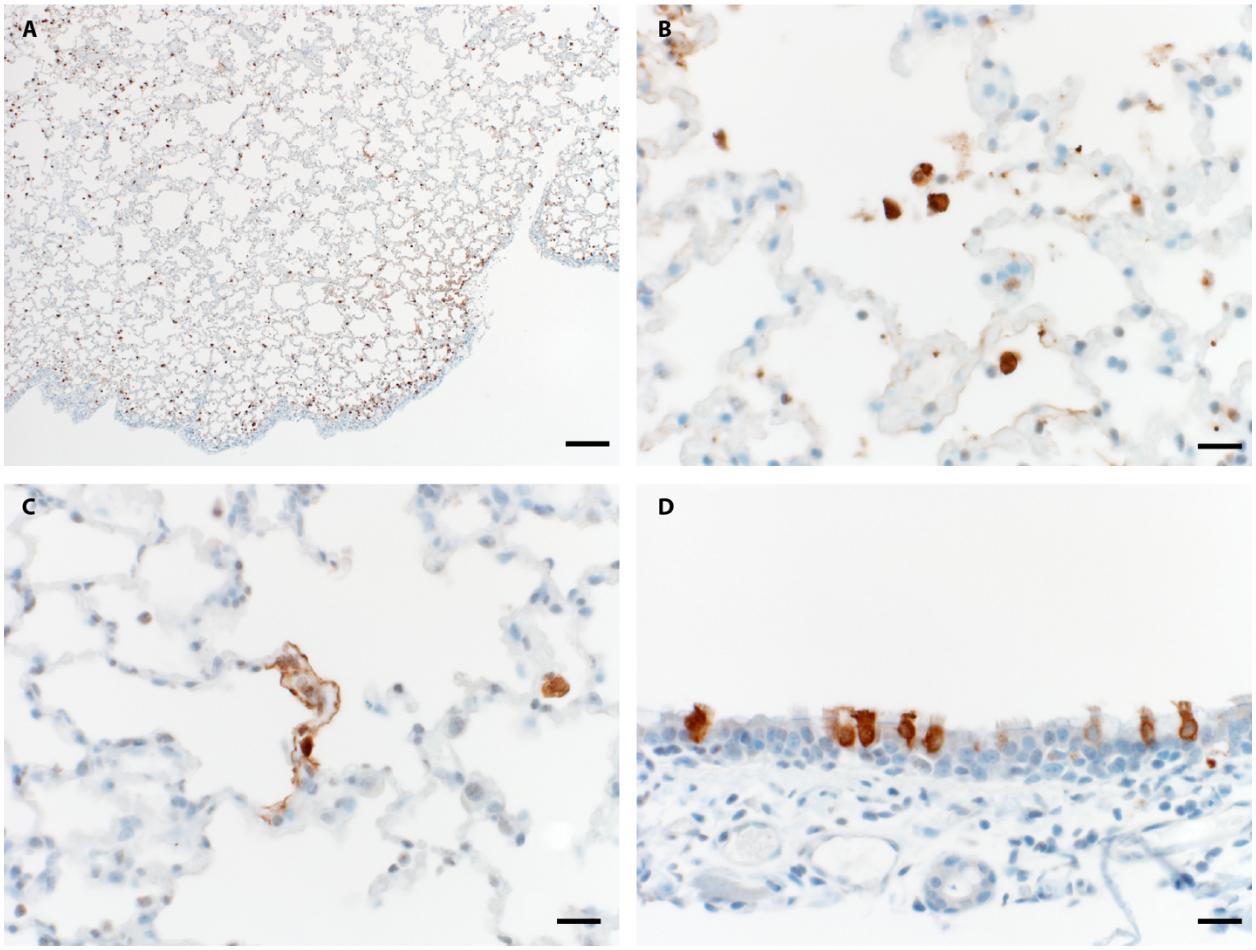

Supplemental Figure 5. SARS-CoV-2 Pulmonary immunohistochemistry. (A) Lung: Bar=200um (B) Alveolar macrophage immunoreactivity (C) Type I & II pneumocyte immunoreactivity (D) Bronchiolar epithelium immunoreactivity (brown=immunoreactive cells) C-E Bar=20um

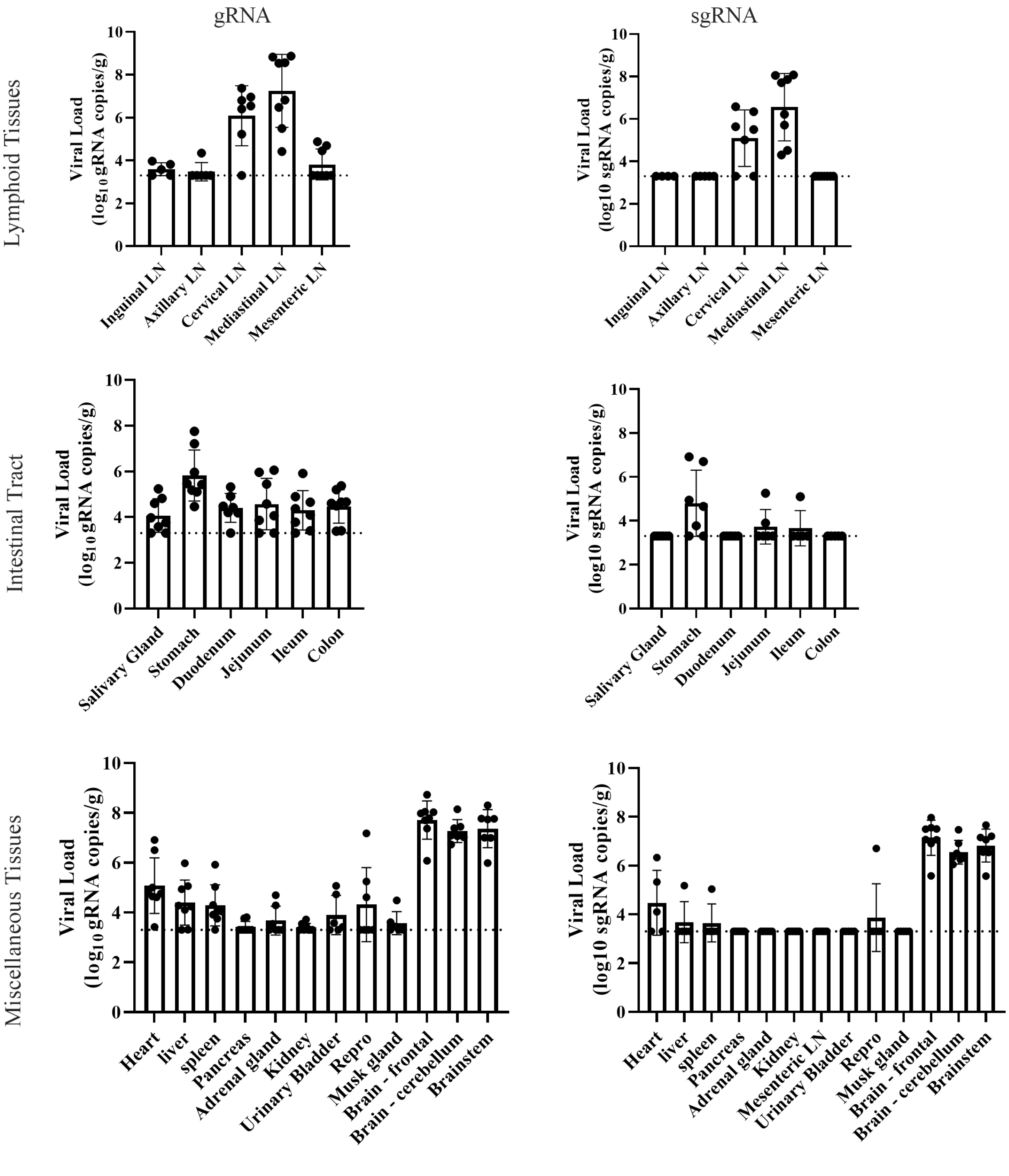

Supplemental Figure 6. Viral burden in non-respiratory tissues. Lymphoid (A), intestinal (B), and miscellaneous (C) tissues from animals euthanized on 3 DPI were evaluated for genomic and sub-genomic RNA. Graphs depict the mean and standard deviation.

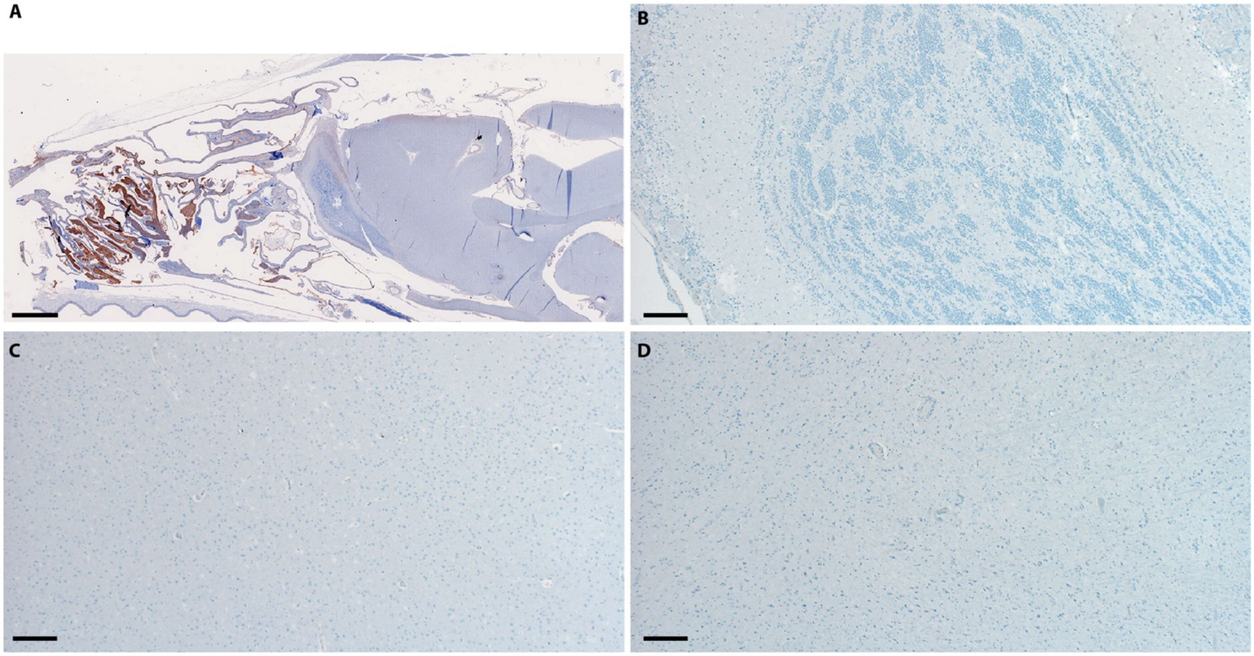

Supplemental Figure 7. Nasal turbinate and brain SARS-CoV-2 immunohistochemistry. (A) Sagittal section of skull: Abundant nasal turbinate epithelium and exudate immunoreactivity (brown); bar = 3mm (B) Olfactory bulb: no immunoreactivity (C) Cerebral cortex: no immunoreactivity (D) Brainstem: no immunoreactivity. B-D bar = 200µm

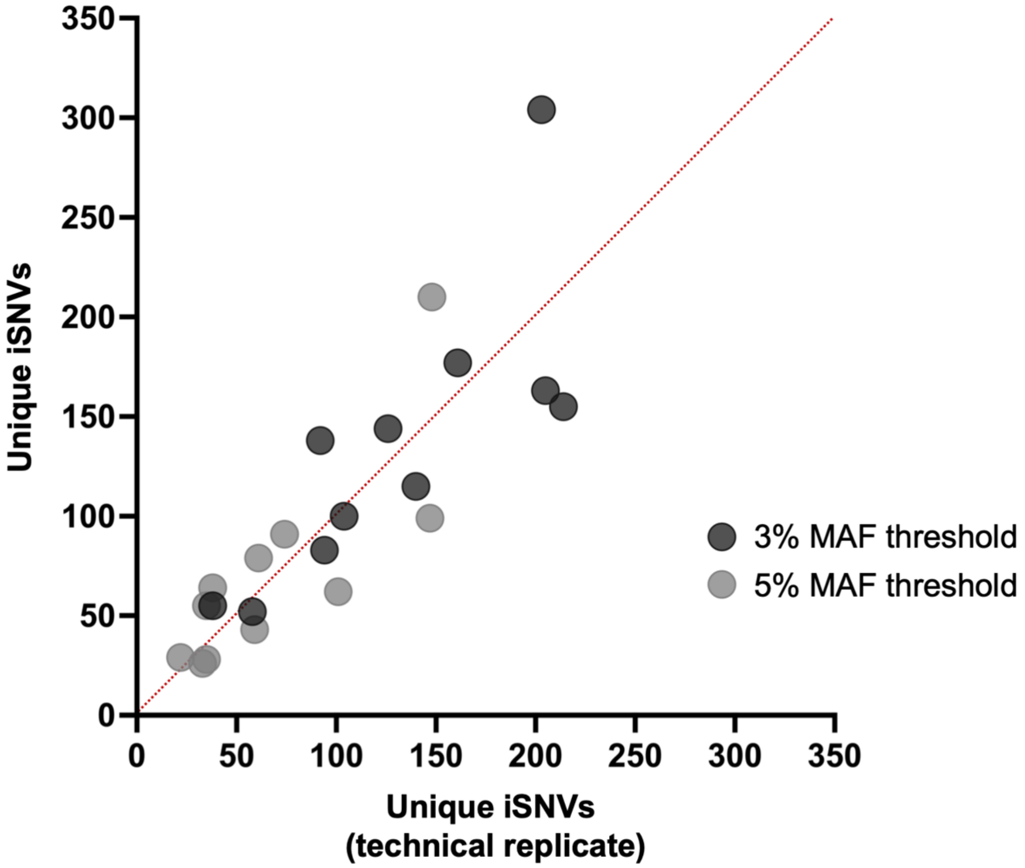

Supplemental Figure 8. **Comparison of intrahost single nucleotide variants (iSNVs) detected between technical replicates at minor allele frequencies (MAFs) of 3% and 5%.** Dots along the red line indicate perfect concordance between replicates, whereas dots farther from the red line indicate less concordance between replicates.

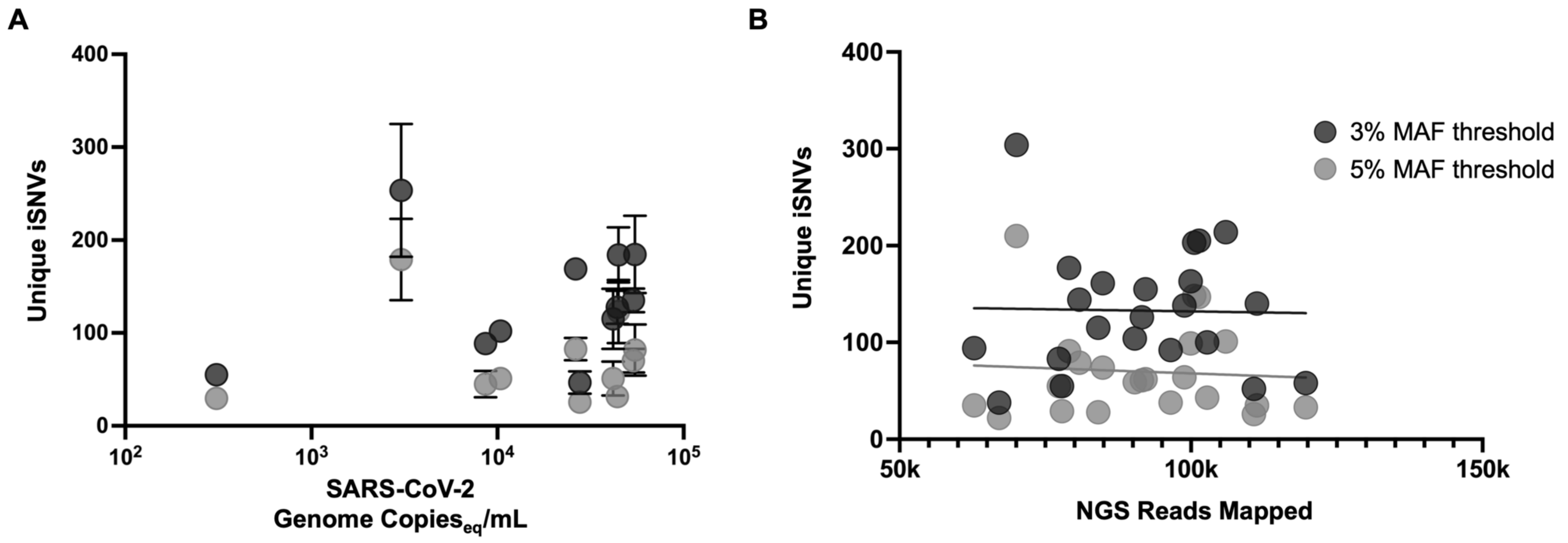

Supplemental Figure 9 (A) Unique intrahost single nucleotide variants (iSNVs) relative to SARS-CoV-2 genome copy number or (B) number of sequenced reads mapped for each sample. Data shown for all samples with minor allele frequency thresholds of 3% and 5%.

Name	Days Post Inoculation	Sample Type	NSP5 (Ct)	SARS-CoV-2 genome copies (eq)/mL	Reads Mapped	Reads Mapped (Technical Rep)
mink01	1	oral swab	25.2	1.82E+05	82,994	
	3	lung tissue	24.8	2.34E+05	96,688	
	3	oral swab	25.4	1.59E+05	73,964	
mink02	1	oral swab	23.5	5.48E+05	94,620	
	2	lung tissue	20.5	3.53E+06	83,812	
	2	oral swab	27.1	5.48E+04	92,177	105,980
mink03	1	oral swab	27.1	5.37E+04	80,835	91,544
	3	lung tissue	20.5	3.72E+06	68,368	
	3	oral swab	25	2.08E+05	87,343	
mink04	1	oral swab	23.9	4.16E+05	89,083	
	3	lung tissue	17.9	1.90E+07	120,414	
	3	oral swab	24.1	3.72E+05	90,275	
mink05	1	oral swab	27.5	4.18E+04	98,844	96,509
	3	lung tissue	17.6	2.28E+07	95,960	
	3	oral swab	25.1	1.91E+05	82,691	
mink06	1	oral swab	24.9	2.12E+05	82,531	
	3	lung tissue	16.9	3.59E+07	83,560	
	3	oral swab	31.6	3.04E+03	70,069	100,539
mink07	1	oral swab	23.1	6.73E+05	82,750	
	2	lung tissue	25.2	1.84E+05	83,869	
	2	oral swab	29.7	1.04E+04	102,762	90,328
mink08	1	oral swab	25.8	1.24E+05	96,249	
	3	lung tissue	16.6	4.44E+07	91,707	
mink09	1	oral swab	26	1.10E+05	105,882	
	3	lung tissue	16.1	5.93E+07	70,836	
	3	oral swab	27.4	4.47E+04	99,943	101,347
mink10	1	oral swab	27.8	3.32E+04	77,189	13 (excluded)
	3	lung tissue	28.1	2.77E+04	77,862	67,088
	3	oral swab	27.4	4.41E+04	84,064	111,319
mink11 (survivor)	1	oral swab	24.9	2.14E+05	66,375	
	3	oral swab	28.2	2.63E+04	79,066	84,833
	5	oral swab	35.2	3.09E+02	110,791	119,696
	7	oral swab	30	8.64E+03	77,324	62,811
Group 1	0	inoculum	24.7	2.50E+05	75,081	
Group 2	0	inoculum	24.2	3.34E+05	91,597	

Supplemental Table 1. **Samples from mink experimentally challenged with SARS-CoV-2 that were deep sequenced for within-host evolutionary analyses.**

Supplemental Table 2. Acknowledgements for mink-associated sequences downloaded from GISAID.

We gratefully acknowledge the following Authors from the Originating laboratories responsible for obtaining the specimens, as well as the Submitting laboratories where the genome data were generated and shared via GISAID, on which this research is based.

All Submitters of data may be contacted directly via www.gisaid.org

Authors are sorted alphabetically.

Accession ID	Originating Laboratory	Submitting Laboratory	Authors
EPI_ISL_717714, EPI_ISL_717715, EPI_ISL_717716, EPI_ISL_717717, EPI_ISL_717718	Animal Health Centre, British Columbia Ministry of Agriculture	National Centre for Foreign Animal Disease, Canadian Food Inspection Agency	Asma Sultana; Brad Pickering; Oliver Lung; Peter Kruczkiewicz; Tomy Joseph
EPI_ISL_2141680, EPI_ISL_2141681, EPI_ISL_2141682, EPI_ISL_2141683, EPI_ISL_2141684, EPI_ISL_2141685, EPI_ISL_2141686, EPI_ISL_2141761, EPI_ISL_2141762, EPI_ISL_2141898, EPI_ISL_2141899, EPI_ISL_2141900, EPI_ISL_2141901, EPI_ISL_2141902, EPI_ISL_2141903, EPI_ISL_2141904, EPI_ISL_2141905, EPI_ISL_2141906, EPI_ISL_2141907, EPI_ISL_2141908, EPI_ISL_2141909, EPI_ISL_2141910, EPI_ISL_4548647, EPI_ISL_4548648, EPI_ISL_4548649, EPI_ISL_4548650, EPI_ISL_8174953, EPI_ISL_8174954, EPI_ISL_8174955, EPI_ISL_8175130, EPI_ISL_8175132, EPI_ISL_8175133, EPI_ISL_8514993, EPI_ISL_8514994, EPI_ISL_8514995, EPI_ISL_8514996, EPI_ISL_8514997, EPI_ISL_8527347, EPI_ISL_8527348, EPI_ISL_8527349, EPI_ISL_8527350, EPI_ISL_8527351, EPI_ISL_8527352, EPI_ISL_8527353, EPI_ISL_8619819, EPI_ISL_8619820, EPI_ISL_8619821, EPI_ISL_8619822, EPI_ISL_8619823, EPI_ISL_8619824, EPI_ISL_8619825, EPI_ISL_8619826, EPI_ISL_8619827, EPI_ISL_8619828, EPI_ISL_8619829, EPI_ISL_8619830, EPI_ISL_8619831			
see above	BIOR	Latvian Biomedical Research and Study Centre	Alise Jakovele; Daina Pule; Davids Fridmanis; Elina Dimina; Guntars Zarins; Irena Meistere; Ivars Silamikelis; Janis Klovins; Janis Pjalkovskis; Jurijs Perevoscikovs; Kaspars Megnis; Laila Silamikele; Lauma Freimane; Laura Ansone; Liga Birzniece; Monta Briviba; Monta Ustinova; Nikita Zrelovs; Uga Dumpis; Una Krumina; Vita Rovite
EPI_ISL_6885298, EPI_ISL_6885299, EPI_ISL_6885300, EPI_ISL_6885301, EPI_ISL_6885302	Centro de Encefalopatías y Enfermedades Transmisibles Emergentes, Universidad de Zaragoza	Instituto de Biomedicina de Valencia (CSIC)	Alicia Otero; Belén Marín; Bernardino Moreno; Cristina Acín; Diego Sola; Eloísa Sevilla; Eva Monleón; Irving Cancino-Muñoz; Iñaki Comas; Juan José Badiola Díez; Marina Betancor; Marta Monzón; Mirta García Martínez; Rosa Bolea; Santiago Jiménez-Serrano; Sonia Pérez Lázaro
EPI_ISL_7721854	Departament of Virology National Veterinary Research Institute	Department of Omics Analysis National Veterinary Research Institute	Bomba Arkadiusz; Domanska-Blicharz Katarzyna; Iwan Ewelina; Lisowska Anna; Niemczuk Krzysztof; Opolska Justyna; Orlowska Anna; Rola Jerzy; Smreczak Marcin; Trebas Pawel
EPI_ISL_984305, EPI_ISL_984307	Department of Poultry Diseases	Department of Omics Analysis	Arkadiusz Bomba; Domanska-Blicharz Katarzyna; Ewelina Iwan; Niemczuk Krzysztof; Orlowska Anna; Smreczak Marcin
EPI_ISL_732948, EPI_ISL_732949, EPI_ISL_732950, EPI_ISL_732951, EPI_ISL_732952, EPI_ISL_732953, EPI_ISL_732954, EPI_ISL_732955, EPI_ISL_732956, EPI_ISL_732957, EPI_ISL_732958, EPI_ISL_732959			
see above	Department of Tropical Parasitology	Laboratory of Recombinant Vaccines	Boguslaw Szewczyk; Kirsi Aaltonen; Lukasz Rabalski; Maciej Grzybek; Maciej Kosinski; Ravi Kant; Tarja Sironen; Teemu Smura
EPI_ISL_8693816, EPI_ISL_8693906, EPI_ISL_8693911, EPI_ISL_8693912, EPI_ISL_8693913, EPI_ISL_8693914, EPI_ISL_8693915			
see above	Department of Virology, National Veterinary Research Institute	Department of Omics Analysis, National Veterinary Research Institute	Bomba Arkadiusz; Domanska-Blicharz Katarzyna; Iwan Ewelina; Lisowska Anna; Niemczuk Krzysztof; Opolska Justyna; Orlowska Anna; Rola Jerzy; Smreczak Marcin; Trebas Pawel
EPI_ISL_3218555, EPI_ISL_3218557	Department of Virology, National Veterinary Research Institute	Erasmus Medical Center	Anne van der Linden; Annemiek van der Eijk; Bas Oude Munnink; Corine GeurtsvanKessel; David Nieuwenhuijse; Domanska-Blicharz Katarzyna; Emmanuelle Munger; Irina Chestakova; Marion Koopmans; Marjan Boter; Niemczuk Krzysztof; Orlowska Anna; Reina Sikkema; Richard Molenkamp; Rola Jerzy; Smreczak Marcin; on behalf of the Dutch national COVID-19 response team.
EPI_ISL_683005, EPI_ISL_683006, EPI_ISL_683007, EPI_ISL_683008, EPI_ISL_683009, EPI_ISL_683010, EPI_ISL_683011, EPI_ISL_683012, EPI_ISL_683013, EPI_ISL_683014, EPI_ISL_683015, EPI_ISL_683016, EPI_ISL_683017, EPI_ISL_683018, EPI_ISL_683019, EPI_ISL_683020, EPI_ISL_683021, EPI_ISL_683022, EPI_ISL_683023, EPI_ISL_683024, EPI_ISL_683025, EPI_ISL_683026, EPI_ISL_683027, EPI_ISL_683028, EPI_ISL_683029, EPI_ISL_683030, EPI_ISL_683031, EPI_ISL_683032, EPI_ISL_683033, EPI_ISL_683034, EPI_ISL_683035, EPI_ISL_683036, EPI_ISL_683037, EPI_ISL_683038, EPI_ISL_683039, EPI_ISL_683040, EPI_ISL_683041, EPI_ISL_683042, EPI_ISL_683043, EPI_ISL_683044, EPI_ISL_683045, EPI_ISL_683046, EPI_ISL_683047, EPI_ISL_683048, EPI_ISL_683049, EPI_ISL_683050, EPI_ISL_683051, EPI_ISL_683052, EPI_ISL_683053, EPI_ISL_683054, EPI_ISL_683055, EPI_ISL_683056, EPI_ISL_683057, EPI_ISL_683058, EPI_ISL_683059, EPI_ISL_683060, EPI_ISL_683061, EPI_ISL_683062, EPI_ISL_683063, EPI_ISL_683064, EPI_ISL_683065, EPI_ISL_683066, EPI_ISL_683067, EPI_ISL_683068, EPI_ISL_683069, EPI_ISL_683070, EPI_ISL_683071, EPI_ISL_683072, EPI_ISL_683073, EPI_ISL_683074, EPI_ISL_683075, EPI_ISL_683076, EPI_ISL_683077, EPI_ISL_683078, EPI_ISL_683079, EPI_ISL_683080, EPI_ISL_683081, EPI_ISL_683082, EPI_ISL_683083, EPI_ISL_683084, EPI_ISL_683085, EPI_ISL_683086, EPI_ISL_683087, EPI_ISL_683088, EPI_ISL_683089, EPI_ISL_683090, EPI_ISL_683091, EPI_ISL_683092, EPI_ISL_683093, EPI_ISL_683094, EPI_ISL_683095, EPI_ISL_683096, EPI_ISL_683097, EPI_ISL_683098, EPI_ISL_683099, EPI_ISL_683100, EPI_ISL_683101, EPI_ISL_683102, EPI_ISL_683103, EPI_ISL_683104, EPI_ISL_683105, EPI_ISL_683106, EPI_ISL_683107, EPI_ISL_683108, EPI_ISL_683109, EPI_ISL_683110, EPI_ISL_683111, EPI_ISL_683112, EPI_ISL_683113, EPI_ISL_683114, EPI_ISL_683115, EPI_ISL_683116, EPI_ISL_683117, EPI_ISL_683118, EPI_ISL_683119, EPI_ISL_683120, EPI_ISL_683121, EPI_ISL_683122, EPI_ISL_683123, EPI_ISL_683124, EPI_ISL_683125, EPI_ISL_683126, EPI_ISL_683127, EPI_ISL_683128, EPI_ISL_683129, EPI_ISL_683130, EPI_ISL_683131, EPI_ISL_683132, EPI_ISL_683133, EPI_ISL_683134, EPI_ISL_683135, EPI_ISL_683136, EPI_ISL_683137, EPI_ISL_683138, EPI_ISL_683139, EPI_ISL_683140, EPI_ISL_683141, EPI_ISL_683142, EPI_ISL_683143, EPI_ISL_683144, EPI_ISL_683145, EPI_ISL_683146, EPI_ISL_683147, EPI_ISL_683148, EPI_ISL_683149, EPI_ISL_683150, EPI_ISL_683151, EPI_ISL_683152, EPI_ISL_683153, EPI_ISL_683154, EPI_ISL_683155, EPI_ISL_683156, EPI_ISL_683157, EPI_ISL_683158, EPI_ISL_683159, EPI_ISL_683160, EPI_ISL_683161, EPI_ISL_683162, EPI_ISL_683163, EPI_ISL_683167, EPI_ISL_683168, EPI_ISL_683169, EPI_ISL_683170, EPI_ISL_683171, EPI_ISL_683172, EPI_ISL_683173, EPI_ISL_683174, EPI_ISL_683175, EPI_ISL_683176, EPI_ISL_683177, EPI_ISL_683178, EPI_ISL_683179, EPI_ISL_683180, EPI_ISL_683181, EPI_ISL_683182, EPI_ISL_683183, EPI_ISL_683184, EPI_ISL_683185, EPI_ISL_683186, EPI_ISL_683187, EPI_ISL_683188, EPI_ISL_683189, EPI_ISL_683190, EPI_ISL_683191, EPI_ISL_683192, EPI_ISL_683193, EPI_ISL_683194, EPI_ISL_683195, EPI_ISL_683196, EPI_ISL_683197, EPI_ISL_683198, EPI_ISL_683199, EPI_ISL_683200, EPI_ISL_683201, EPI_ISL_683202, EPI_ISL_683203, EPI_ISL_683204, EPI_ISL_683205, EPI_ISL_683206, EPI_ISL_683207, EPI_ISL_683208, EPI_ISL_683209, EPI_ISL_683210, EPI_ISL_683211, EPI_ISL_683212, EPI_ISL_683213, EPI_ISL_683214, EPI_ISL_683215, EPI_ISL_683216, EPI_ISL_683217, EPI_ISL_683218, EPI_ISL_683219, EPI_ISL_683220, EPI_ISL_683221, EPI_ISL_683222, EPI_ISL_683223, EPI_ISL_683224, EPI_ISL_683225, EPI_ISL_683226, EPI_ISL_683227, EPI_ISL_683228, EPI_ISL_683229, EPI_ISL_683230, EPI_ISL_683231, EPI_ISL_683232, EPI_ISL_683233, EPI_ISL_683234, EPI_ISL_683235, EPI_ISL_683236, EPI_ISL_683237, EPI_ISL_683238, EPI_ISL_683239, EPI_ISL_683240, EPI_ISL_683241, EPI_ISL_683242, EPI_ISL_683243, EPI_ISL_683244, EPI_ISL_683245, EPI_ISL_683246, EPI_ISL_683247, EPI_ISL_683248, EPI_ISL_683249, EPI_ISL_683250, EPI_ISL_683251, EPI_ISL_683252, EPI_ISL_683253, EPI_ISL_683254, EPI_ISL_683255, EPI_ISL_683256, EPI_ISL_683257, EPI_ISL_683258, EPI_ISL_683259, EPI_ISL_683260, EPI_ISL_683261, EPI_ISL_683262, EPI_ISL_683263, EPI_ISL_683264, EPI_ISL_683265, EPI_ISL_683266, EPI_ISL_683267, EPI_ISL_683268, EPI_ISL_683269, EPI_ISL_683270, EPI_ISL_683271, EPI_ISL_683272, EPI_ISL_683273, EPI_ISL_683274, EPI_ISL_683275, EPI_ISL_683276, EPI_ISL_683277, EPI_ISL_683278, EPI_ISL_683279, EPI_ISL_683280, EPI_ISL_683281, EPI_ISL_683282, EPI_ISL_683283, EPI_ISL_683284, EPI_ISL_683285, EPI_ISL_683286, EPI_ISL_683287, EPI_ISL_683288, EPI_ISL_683289, EPI_ISL_683290, EPI_ISL_683291, EPI_ISL_683292, EPI_ISL_683293, EPI_ISL_683294, EPI_ISL_683295, EPI_ISL_683296, EPI_ISL_683297, EPI_ISL_683298, EPI_ISL_683299, EPI_ISL_683300, EPI_ISL_683301, EPI_ISL_683302, EPI_ISL_683303, EPI_ISL_683304, EPI_ISL_683305, EPI_ISL_683306, EPI_ISL_683307, EPI_ISL_683308, EPI_ISL_683309, EPI_ISL_683310, EPI_ISL_683311, EPI_ISL_683312, EPI_ISL_683313, EPI_ISL_683314, EPI_ISL_683315, EPI_ISL_683316, EPI_ISL_683317, EPI_ISL_683318, EPI_ISL_683319, EPI_ISL_683320, EPI_ISL_683321, EPI_ISL_683322, EPI_ISL_683323, EPI_ISL_683324, EPI_ISL_683325, EPI_ISL_683326, EPI_ISL_683327, EPI_ISL_683328			
see above	Department of Virus and Microbiological Special Diagnostics, Statens Serum Institut, Copenhagen, Denmark	Albertsen Lab, Department of Chemistry and Bioscience, Aalborg University, Denmark	Danish Covid-19 Genome Consortium
EPI_ISL_641396, EPI_ISL_641397, EPI_ISL_641398, EPI_ISL_641399, EPI_ISL_641400, EPI_ISL_641401, EPI_ISL_641402, EPI_ISL_641403, EPI_ISL_641404, EPI_ISL_641405, EPI_ISL_641406, EPI_ISL_641407, EPI_ISL_641408, EPI_ISL_641409, EPI_ISL_641410, EPI_ISL_641411, EPI_ISL_641412, EPI_ISL_641413, EPI_ISL_641414, EPI_ISL_641415, EPI_ISL_641416, EPI_ISL_641417, EPI_ISL_641418, EPI_ISL_641419, EPI_ISL_641420, EPI_ISL_641421, EPI_ISL_641422, EPI_ISL_641423, EPI_ISL_641424, EPI_ISL_641425, EPI_ISL_641426, EPI_ISL_641427, EPI_ISL_641428, EPI_ISL_641429, EPI_ISL_641430, EPI_ISL_641431, EPI_ISL_641432, EPI_ISL_641433, EPI_ISL_641434, EPI_ISL_641435, EPI_ISL_641436, EPI_ISL_641437, EPI_ISL_641438, EPI_ISL_641439, EPI_ISL_641440, EPI_ISL_641441, EPI_ISL_641442, EPI_ISL_641443, EPI_ISL_641444, EPI_ISL_641445, EPI_ISL_641446, EPI_ISL_641447, EPI_ISL_641448, EPI_ISL_641449, EPI_ISL_641450, EPI_ISL_641451, EPI_ISL_641452, EPI_ISL_641453, EPI_ISL_641454, EPI_ISL_641455, EPI_ISL_641456, EPI_ISL_641457, EPI_ISL_641458, EPI_ISL_641459, EPI_ISL_641460, EPI_ISL_641461, EPI_ISL_641462, EPI_ISL_641463, EPI_ISL_641464, EPI_ISL_641465, EPI_ISL_641466, EPI_ISL_641467, EPI_ISL_641468, EPI_ISL_641469, EPI_ISL_641470, EPI_ISL_641471, EPI_ISL_641472, EPI_ISL_641473, EPI_ISL_641474, EPI_ISL_641475, EPI_ISL_641476, EPI_ISL_641477, EPI_ISL_641478, EPI_ISL_641479, EPI_ISL_641480, EPI_ISL_641481, EPI_ISL_641482, EPI_ISL_641483, EPI_ISL_641484, EPI_ISL_641485, EPI_ISL_641486, EPI_ISL_641487, EPI_ISL_641488, EPI_ISL_641489, EPI_ISL_641490, EPI_ISL_641491, EPI_ISL_641492, EPI_ISL_641493, EPI_ISL_641494, EPI_ISL_641495, EPI_ISL_641496, EPI_ISL_641497, EPI_ISL_641498, EPI_ISL_641499, EPI_ISL_641500, EPI_ISL_641501, EPI_ISL_641502, EPI_ISL_641503, EPI_ISL_641504, EPI_ISL_641505, EPI_ISL_641506, EPI_ISL_641507, EPI_ISL_641508, EPI_ISL_641509, EPI_ISL_641510, EPI_ISL_641511, EPI_ISL_641512, EPI_ISL_641513, EPI_ISL_641514, EPI_ISL_641515, EPI_ISL_641516, EPI_ISL_3050753, EPI_ISL_3050754, EPI_ISL_3050755, EPI_ISL_3050756, EPI_ISL_3050757, EPI_ISL_3050758, EPI_ISL_3050759, EPI_ISL_3050760, EPI_ISL_3050761, EPI_ISL_3050762, EPI_ISL_3050763, EPI_ISL_3050764, EPI_ISL_3050765, EPI_ISL_3050766			
see above	Department of Virus and Microbiological Special Diagnostics, Statens Serum Institut, Copenhagen, Denmark	Albertsen lab, Department of Chemistry and Bioscience, Aalborg University, Denmark	Danish Covid-19 Genome Consortia; Jannik Fonager; Morten Rasmussen; Thomas Bruun Rasmussen
EPI_ISL_522987, EPI_ISL_522988, EPI_ISL_522989, EPI_ISL_522990, EPI_ISL_522991, EPI_ISL_522992, EPI_ISL_522993, EPI_ISL_522994, EPI_ISL_522995, EPI_ISL_522996, EPI_ISL_522997, EPI_ISL_522998, EPI_ISL_522999, EPI_ISL_523000, EPI_ISL_523001, EPI_ISL_523002, EPI_ISL_523003, EPI_ISL_523004, EPI_ISL_523005, EPI_ISL_523006, EPI_ISL_523007, EPI_ISL_523008, EPI_ISL_523009, EPI_ISL_523010, EPI_ISL_523011, EPI_ISL_523012, EPI_ISL_523013, EPI_ISL_523014, EPI_ISL_523015, EPI_ISL_523016, EPI_ISL_523017, EPI_ISL_523018, EPI_ISL_523019, EPI_ISL_523020, EPI_ISL_523021, EPI_ISL_523022, EPI_ISL_523023, EPI_ISL_523024, EPI_ISL_523025, EPI_ISL_523026, EPI_ISL_523027, EPI_ISL_523028, EPI_ISL_523029, EPI_ISL_523030, EPI_ISL_523031, EPI_ISL_523032, EPI_ISL_523033, EPI_ISL_523034, EPI_ISL_523035, EPI_ISL_523036, EPI_ISL_523037, EPI_ISL_523038, EPI_ISL_523039, EPI_ISL_523040, EPI_ISL_523041, EPI_ISL_523042, EPI_ISL_523043, EPI_ISL_523044, EPI_ISL_523045, EPI_ISL_523046, EPI_ISL_523047, EPI_ISL_523048, EPI_ISL_523049, EPI_ISL_523050, EPI_ISL_523051, EPI_ISL_523052, EPI_ISL_523053, EPI_ISL_523054, EPI_ISL_523055, EPI_ISL_523056, EPI_ISL_523057, EPI_ISL_523058, EPI_ISL_523059, EPI_ISL_523060, EPI_ISL_523061, EPI_ISL_523062, EPI_ISL_523063, EPI_ISL_523064, EPI_ISL_523065, EPI_ISL_523066, EPI_ISL_523067, EPI_ISL_523068, EPI_ISL_523069, EPI_ISL_523070, EPI_ISL_523071, EPI_ISL_523072, EPI_ISL_523073, EPI_ISL_523074, EPI_ISL_523075, EPI_ISL_523076, EPI_ISL_523077, EPI_ISL_523078, EPI_ISL_523079, EPI_ISL_523080, EPI_ISL_523081, EPI_ISL_523082, EPI_ISL_523083, EPI_ISL_523084, EPI_ISL_523085, EPI_ISL_523086, EPI_ISL_523087, EPI_ISL_523088, EPI_ISL_523089, EPI_ISL_523090, EPI_ISL_523091, EPI_ISL_523092, EPI_ISL_523093, EPI_ISL_523094, EPI_ISL_523095, EPI_ISL_523096, EPI_ISL_523097, EPI_ISL_523098, EPI_ISL_523099, EPI_ISL_523100, EPI_ISL_523101, EPI_ISL_523102, EPI_ISL_523103, EPI_ISL_523104, EPI_ISL_523105, EPI_ISL_523106, EPI_ISL_523107, EPI_ISL_523108, EPI_ISL_523109, EPI_ISL_523110, EPI_ISL_523111, EPI_ISL_523112, EPI_ISL_523113, EPI_ISL_523114, EPI_ISL_523115, EPI_ISL_523116, EPI_ISL_523117, EPI_ISL_523118, EPI_ISL_523119, EPI_ISL_523120, EPI_ISL_577749, EPI_ISL_577750, EPI_ISL_577751, EPI_ISL_577752, EPI_ISL_577753, EPI_ISL_577754, EPI_ISL_577755, EPI_ISL_577756, EPI_ISL_577757, EPI_ISL_577758, EPI_ISL_577759, EPI_ISL_577760, EPI_ISL_577761, EPI_ISL_577762, EPI_ISL_577763, EPI_ISL_577764, EPI_ISL_577765, EPI_ISL_577766, EPI_ISL_577767, EPI_ISL_577768, EPI_ISL_577769, EPI_ISL_577770, EPI_ISL_577771, EPI_ISL_577772, EPI_ISL_577773, EPI_ISL_577774, EPI_ISL_577775, EPI_ISL_577776, EPI_ISL_577777, EPI_ISL_577778, EPI_ISL_577779, EPI_ISL_577780, EPI_ISL_577781, EPI_ISL_577782, EPI_ISL_577783, EPI_ISL_577784, EPI_ISL_577785, EPI_ISL_577786, EPI_ISL_577787, EPI_ISL_577788, EPI_ISL_577789, EPI_ISL_577790, EPI_ISL_577791, EPI_ISL_577792, EPI_ISL_577793, EPI_ISL_577794, EPI_ISL_577795, EPI_ISL_577796, EPI_ISL_577797, EPI_ISL_577798, EPI_ISL_577799, EPI_ISL_577800, EPI_ISL_577801, EPI_ISL_577802, EPI_ISL_577803, EPI_ISL_577805, EPI_ISL_577807, EPI_ISL_577808, EPI_ISL_577809, EPI_ISL_577810, EPI_ISL_577811, EPI_ISL_577812, EPI_ISL_577813, EPI_ISL_577814, EPI_ISL_577815, EPI_ISL_577816, EPI_ISL_577817, EPI_ISL_577818, EPI_ISL_577819, EPI_ISL_577820, EPI_ISL_577821, EPI_ISL_577822, EPI_ISL_577823, EPI_ISL_577824, EPI_ISL_577825, EPI_ISL_577826, EPI_ISL_577827, EPI_ISL_577828, EPI_ISL_632324, EPI_ISL_632334, EPI_ISL_632348, EPI_ISL_632351, EPI_ISL_632353, EPI_ISL_632380, EPI_ISL_632384, EPI_ISL_632385, EPI_ISL_632396, EPI_ISL_632420, EPI_ISL_632428, EPI_ISL_632429, EPI_ISL_632430, EPI_ISL_632434, EPI_ISL_632435, EPI_ISL_632436, EPI_ISL_632437, EPI_ISL_632438, EPI_ISL_632439, EPI_ISL_632450, EPI_ISL_632451, EPI_ISL_632452, EPI_ISL_632453, EPI_ISL_632454, EPI_ISL_632455, EPI_ISL_632456, EPI_ISL_632457, EPI_ISL_632458, EPI_ISL_632459, EPI_ISL_632462, EPI_ISL_632463, EPI_ISL_632464, EPI_ISL_632465, EPI_ISL_632466, EPI_ISL_632467, EPI_ISL_632468, EPI_ISL_632469, EPI_ISL_632470, EPI_ISL_632471, EPI_ISL_632472, EPI_ISL_632473, EPI_ISL_632474, EPI_ISL_632475, EPI_ISL_632476, EPI_ISL_632477, EPI_ISL_632478, EPI_ISL_632483, EPI_ISL_632484, EPI_ISL_632485, EPI_ISL_632486, EPI_ISL_632487, EPI_ISL_632488, EPI_ISL_632489, EPI_ISL_632490, EPI_ISL_632491, EPI_ISL_632492, EPI_ISL_722320, EPI_ISL_722414, EPI_ISL_722415, EPI_ISL_722416, EPI_ISL_722417, EPI_ISL_722418, EPI_ISL_722448, EPI_ISL_722449, EPI_ISL_722450, EPI_ISL_722451, EPI_ISL_904155, EPI_ISL_904193, EPI_ISL_904320, EPI_ISL_904321, EPI_ISL_904322, EPI_ISL_904323, EPI_ISL_904324, EPI_ISL_904325, EPI_ISL_904326, EPI_ISL_904327, EPI_ISL_904328, EPI_ISL_904329, EPI_ISL_904330, EPI_ISL_904331, EPI_ISL_904332, EPI_ISL_904333, EPI_ISL_904334, EPI_ISL_904335, EPI_ISL_904336, EPI_ISL_904337, EPI_ISL_904338, EPI_ISL_904339, EPI_ISL_904340, EPI_ISL_904341, EPI_ISL_904342, EPI_ISL_904343, EPI_ISL_904344, EPI_ISL_904345, EPI_ISL_904346, EPI_ISL_904347, EPI_ISL_904348, EPI_ISL_904349, EPI_ISL_904350, EPI_ISL_904351, EPI_ISL_904352, EPI_ISL_904353, EPI_ISL_904354, EPI_ISL_904355, EPI_ISL_904356, EPI_ISL_904357, EPI_ISL_904358, EPI_ISL_904359, EPI_ISL_904360, EPI_ISL_904625			
see above	Dutch COVID-19 response team	Erasmus Medical Center	Anne van der Linden; Annemiek van der Eijk; Bas Oude Munnink; Corine GeurtsvanKessel; David Nieuwenhuijse; Emmanuelle Munger; Irina Chestakova; Marion Koopmans; Marjan Boter; OH consortium; Reina Sikkema; Richard Molenkamp; on behalf of the OH consortium.
EPI_ISL_1265354, EPI_ISL_1265355, EPI_ISL_1265356, EPI_ISL_1265357, EPI_ISL_1265358, EPI_ISL_1265359, EPI_ISL_1265360, EPI_ISL_1265361, EPI_ISL_1265362, EPI_ISL_1265363, EPI_ISL_1265364, EPI_ISL_1265365, EPI_ISL_1265366, EPI_ISL_1265367			
see above	Dutch COVID-19 response team	Wageningen Bioveterinary Research	OH consortium
EPI_ISL_8144460, EPI_ISL_8144461, EPI_ISL_8144462, EPI_ISL_8527354	E. Gulbja Laboratorija	Latvian Biomedical Research and Study Centre	Alise Jakovele; Daina Pule; Davids Fridmanis; Dmitrijs Perminovs; Elina Dimina; Guntars Zarins; Irena Meistere; Ivars Silamikelis; Janis Klovins; Janis Pjalkovskis; Jurijs Perevoscikovs; Kaspars Megnis; Laila Silamikele; Lauma Freimane; Laura Ansone; Liga Birzniece; Mikus Gavars; Monta Briviba; Nikita Zrelovs; Uga Dumpis; Una Krumina; Vita Rovite
EPI_ISL_1392906	Laboratoire de la rage et de la faune sauvage, ANSES, Malzévillle.	GRAM2.0, Université de Caen Normandie / Laboratoire de Virologie, CHU de Caen / National Reference Center for Respiratory Viruses France Nord, Institut Pasteur, Paris. / Laboratoire de la rage et de la faune sauvage ANSES, Malzéville.	Angela BRISEBARRE; Astrid VABRET; Camille Capel; Elodie MONCHATRE-LEROY.; Estelle LEPERCHOIS; Etienne SIMON-LORIERE; Evelyne PICARD-MEYER; Fabiana GAMBARO; Flora DONATI; Franck BOUE; Gilles SALVAT; Januel VOUTEAU; Justine HAMEL; Marine WASNIEWSKY; Maud Vanpeene; Meriadeg AR GOUILH; Mélanie ALBERT; Méline Bizard; Nicolas NESI; Sandrine CORBET; Sophie LEVALTIER; Sylvie BEHILLIL; Sylvie VAN DER WERF; Vincent ENOUF
EPI_ISL_925307, EPI_ISL_925308, EPI_ISL_925309, EPI_ISL_925310, EPI_ISL_925311, EPI_ISL_925312, EPI_ISL_925313, EPI_ISL_925314, EPI_ISL_925315, EPI_ISL_925316, EPI_ISL_925317, EPI_ISL_925318, EPI_ISL_925319, EPI_ISL_925320, EPI_ISL_925321, EPI_ISL_925322, EPI_ISL_925323, EPI_ISL_925324, EPI_ISL_925325, EPI_ISL_925326, EPI_ISL_925327, EPI_ISL_925328, EPI_ISL_925329, EPI_ISL_925330, EPI_ISL_925331, EPI_ISL_925332, EPI_ISL_925333, EPI_ISL_925334, EPI_ISL_925335, EPI_ISL_925336, EPI_ISL_925337, EPI_ISL_925338, EPI_ISL_925339, EPI_ISL_925340, EPI_ISL_925341, EPI_ISL_925342, EPI_ISL_925343, EPI_ISL_925344, EPI_ISL_925345, EPI_ISL_925346, EPI_ISL_925347, EPI_ISL_925348, EPI_ISL_925349, EPI_ISL_925350, EPI_ISL_925351, EPI_ISL_925352, EPI_ISL_925353, EPI_ISL_925354, EPI_ISL_925355, EPI_ISL_925356, EPI_ISL_925357, EPI_ISL_925358, EPI_ISL_925359, EPI_ISL_925360			
see above	Michigan Department of Agriculture and Rural Development	Pathogen Discovery, Respiratory Viruses Branch, Division of Viral Diseases, Centers for Disease Control and Prevention	Anna Uehara; Anna montmayeur; Brian Lynch; Clinton R. Paden; Haibin Wang; Jing Zhang; Krista Queen; Peter Cook; Rachel Marine; Suxiang Tong; Yan Li; Ying Tao
EPI_ISL_2834696, EPI_ISL_2834697	Michigan State University Veterinary Diagnostic Laboratory	Diagnostic Virology Laboratory, National Veterinary Services Laboratories, USDA1920 Dayton Avenue, Ames, IA 50010, USA	Emily R. Love; Kerrie M. Franzen; Mary L. Killian; Mia Torchetti; Suelee Robbe-Austerman; Tod P. Stuber
EPI_ISL_851055, EPI_ISL_851056, EPI_ISL_851057, EPI_ISL_851058, EPI_ISL_851059, EPI_ISL_851060, EPI_ISL_851061			
see above	National Food and Veterinary Risk Assessment Institute	Lithuanian University of Health Sciences, Molecular cardiology lab.	Algirdas Malakauskas; Arnoldas Pautienius; Arunas Stankevicius; Astra Vitkauskiene; Dovydas Gecys; Ingrida Olendraitė; Kamile Tamusauskaite; Laura Pareckaite; Lukas Zemaitis; Vaiva Lesauskaite
EPI_ISL_1527630, EPI_ISL_1527631, EPI_ISL_1527632, EPI_ISL_1527633	Oregon Department of Agriculture	Diagnostic Virology Laboratory, National Veterinary Services Laboratories, USDA1920 Dayton Avenue, Ames, IA 50010, USA	Emily R. Love; Kerrie M. Franzen; Mary L. Killian; Mia Torchetti; Suelee Robbe-Austerman; Tod P. Stuber
EPI_ISL_1490748	Pharmacy Department, Facultad de CC Salud, UCH-CEU University	Pharmacy Department, Facultad de CC Salud, UCH-CEU University	Miguel Padilla-Blanco
EPI_ISL_626340, EPI_ISL_626341, EPI_ISL_626342, EPI_ISL_626343, EPI_ISL_626344, EPI_ISL_626345, EPI_ISL_626346, EPI_ISL_626347, EPI_ISL_626348, EPI_ISL_626349, EPI_ISL_626350, EPI_ISL_626351			
see above	Statens Serum Institute	Statens Serum Institute	A.S.; Afaro-Nunez, A.; Belsham; C.S.; Fonager, J.; Foomsgaard, A.; G.J. and Botner, A.; Halasa, T.; Hammer; Jorgensen; Lohse, L.; M.L.; M.W.; Mundbjerg, K.; Quaade; Rasmussen; Rasmussen, M.; Rosenstierne; Strandbygaard, B.; T.B.
EPI_ISL_2467943	The Laboratory of Biosafety with Pathogens Collection The Republican Research and Practical Center for Epidemiology	Laboratory for HIV and opportunistic infections diagnosis The Republican Research and Practical Center for Epidemiology and Microbiology (RRPCEM)	Anatoli Krasko; Artur Akhremchuk; Elena Gasich; Ilarion Kupryianav; Iryna Subotsina; Kirill Bulda; Larysa Radzienava; Leonid Valentovich; Olga Klimovich; Olga Saleuskaya; Sergei Semenov

Supplementary Table 3: Long range PCR primers for amplification of ACE2.

Primer	Sequence (5' - 3')
Amplicon 1-F	CCCAACCCAAGTTCAAAGGCTGATGAGAGAGAA
Amplicon 1-R	CTTCATGTTTAGCTATTTGACAAAGGGCTTCTT
Amplicon 2-F	GGACACATCCAGTATGACATGGCATATG
Amplicon 2-R	TGGGGGAGTACAAGATTTAAGAGATTGGGAA
